# Associations between the Objectively Measured Office Environment and Workplace Step Count and Sitting Time: Cross-Sectional Analyses from the Active Buildings Study

**DOI:** 10.3390/ijerph15061135

**Published:** 2018-06-01

**Authors:** Abi Fisher, Marcella Ucci, Lee Smith, Alexia Sawyer, Richard Spinney, Marina Konstantatou, Alexi Marmot

**Affiliations:** 1 Department of Behavioural Science and Health, University College London, 1–19 Torrington Place, London WC1E 6BT, UK; alexia.sawyer@ucl.ac.uk; 2 Bartlett School of Environmental Design and Engineering, UCL Bartlett Faculty of the Built Environment, University College London, Central House, 14 Upper Woburn place, London WC1H 0NN, UK; m.ucci@ucl.ac.uk; 3 The Centre for Sport and Exercise Sciences, Anglia Ruskin University, Cambridge CB1 1PT, UK; Lee.Smith@anglia.ac.uk; 4 Complex Systems Research Group & Centre for Complex Systems, Faculty of Engineering and IT, The University of Sydney, Sydney, NSW 2006, Australia; richard.spinney@sydney.edu.au; 5 Structures Group, University of Cambridge, Trumpington Street, Cambridge CB2 1PZ, UK; mk822@cam.ac.uk; 6 UCL Bartlett Faculty of the Built Environment, University College London, Gordon House, 29 Gordon Square, London WC1H 0PP, UK; a.marmot@ucl.ac.uk

**Keywords:** occupational physical activity, sedentary behaviour, office-based work

## Abstract

Office-based workers spend a large proportion of the day sitting and tend to have low overall activity levels. Despite some evidence that features of the external physical environment are associated with physical activity, little is known about the influence of the spatial layout of the internal environment on movement, and the majority of data use self-report. This study investigated associations between objectively-measured sitting time and activity levels and the spatial layout of office floors in a sample of UK office-based workers. Participants wore activPAL accelerometers for at least three consecutive workdays. Primary outcomes were steps and proportion of sitting time per working hour. Primary exposures were office spatial layout, which was objectively-measured by deriving key spatial variables: ‘distance from each workstation to key office destinations’, ‘distance from participant’s workstation to all other workstations’, ‘visibility of co-workers’, and workstation ‘closeness’. 131 participants from 10 organisations were included. Fifty-four per cent were female, 81% were white, and the majority had a managerial or professional role (72%) in their organisation. The average proportion of the working hour spent sitting was 0.7 (SD 0.15); participants took on average 444 (SD 210) steps per working hour. Models adjusted for confounders revealed significant negative associations between step count and distance from each workstation to all other office destinations (e.g., B = −4.66, 95% CI: −8.12, −1.12, *p* < 0.01) and nearest office destinations (e.g., B = −6.45, 95% CI: −11.88, −0.41, *p* < 0.05) and visibility of workstations when standing (B = −2.35, 95% CI: −3.53, −1.18, *p* < 0.001). The magnitude of these associations was small. There were no associations between spatial variables and sitting time per work hour. Contrary to our hypothesis, the further participants were from office destinations the less they walked, suggesting that changing the relative distance between workstations and other destinations on the same floor may not be the most fruitful target for promoting walking and reducing sitting in the workplace. However, reported effect sizes were very small and based on cross-sectional analyses. The approaches developed in this study could be applied to other office buildings to establish whether a specific office typology may yield more promising results.

## 1. Background

The health benefits of regular and sustained participation in physical activity are well established and include reduced risk of cardiovascular disease (CVD), diabetes, some cancers, mental illness and all-cause mortality [1,2,3]. In addition, unless individuals are accumulating ≤60 min of moderate activity per day, high levels of sitting time can independently negatively impact health [4,5,6,7]. Indeed, a meta-analysis including over 1 million adults suggests that even with this level of activity, the risk from high levels of sedentary behaviour is lessened but not eliminated [8]. However, UK-population levels of activity remain low; accelerometer data in 4507 adults in the Heath Survey for England found that <10% met even minimum Government guidelines for health of ≥150 min of moderate activity per week [9]. Office-based workers have high levels of sitting time, often coupled with low levels of physical activity. Estimates of physical activity levels in office-based workers suggest daily step counts of 4000 to 6000, categorising these individuals as ‘low-active’ [10]. For 131 UK office workers in the Active Buildings study, objectively-measured levels of sedentary time were extremely high in work time [11]. In such low-active individuals, small increases in physical activity could benefit health, so strategies to increase activity and reduce sitting time are required urgently. 

The office environment provides a ‘captive audience’ for activity promotion and has shown promise in previous studies. In a meta-analysis of 138 randomised controlled trials involving 38,231 adults, workplace physical activity interventions significantly increased physical activity [12]. However, the studies included in the review focussed on interventions delivered in the workplace by external parties (e.g., researchers) and were limited by lack of long-term follow up. In addition, these types of interventions are typically oversubscribed by those most motivated to change and therefore neglect those who may benefit most from small increases in activity [12,13]. In addition, little is understood about the type of intervention that can effectively reduce sitting time; a systematic review of six interventions to reduce sitting in the office by targeting individual level factors found small or no effects [14]. One plausible solution is to modify the layout and furniture within the office environment to facilitate movement, and ‘nudge’ people toward increased movement and reduced sitting.

A larger body of literature reveals the influence of the external built environment on physical activity levels. For example, a review of reviews [15] identified that short distance to destinations, attractiveness of the environment, and mixed land use were associated with more outdoor walking. A longer distance from home to work and low street connectivity have been associated with lower levels of activity [16]. Within buildings, beyond point-of-choice motivational signage which results in modest increases in stair use [17,18], far less is known about whether or how the indoor built environment influences physical activity and sitting time, and therefore what might be the best targets for modification. Zimring et al. hypothesised that several aspects of building design may influence physical activity including: selection and design of site, building design (e.g., floor size and geometry), and building ‘elements’ such as the layout of workstations and placement of coffee machines and printers [19]. That review suggested that the next research steps should be to understand such building elements, e.g., through the operationalisation of measures, and explore their associations with movement [19]. 

A study in 307 Australian adults presented the development of a reliable self-report measure of workplace spatial metrics and occupational sedentary behaviour and reported cross-sectional associations between subjective aspects of the office environment and sitting time. In that study, greater visibility of co-workers was associated with higher levels of sitting, and higher reported connectivity was associated with lower levels of sitting [20]. In an opportunistic pre-post study of 42 office workers who were relocating from a 1970s building to a newer building that the authors hypothesised would be more ‘activity permissive’, there was a significant decrease in accelerometer assessed workplace sitting time, an increase in standing time, but a significant decline in workplace moderate activity, in the newer building [21]. Elsewhere, relocation from traditional offices to ‘activity-based’ offices (comprising open plan offices with multiple function-specific rooms) was reported to have no effect on objectively-measured sitting time at a 12-month follow in 110 participants, although a small but significant increase in walking time was revealed [22]. The opportunistic use of the office relocation should be commended in these contexts. However, aside from overall floor space, there was no objective assessment of and adjustment for the built environment pre or post, making it unclear which elements of the new environment impacted activity. In 115 UK adults participating in the Active Buildings study, perceived workplace management discouragement of taking breaks and greater perceived distance to key destinations in the office (kitchens/coffee points, printers, toilets) were associated with lower accelerometer measured workplace step count [23]. However, there remains a need to understand how the objectively-measured spatial layout of offices related to movement of the workers within, in order to identify if there are modifiable elements that could be targeted in interventions.

Therefore, the aim of the current study was to provide the first data on associations between objectively-measured indoor spatial layout, and physical activity/sitting time. We hypothesised that the greater the distance from workstations to key destinations within the office, and the lower the visual connectivity, the more participants would walk and the less they would sit.

## 2. Methods

The Active Buildings protocol has been described previously [24]. Active Buildings is an exploratory cross-sectional study examining associations between objectively measured office layout and objectively measured workplace step count and sitting time. Organisations in London and South East UK were contacted via email, and preliminary meetings were held with their Occupational Health (or other relevant) Departments. Once an organisation agreed to participate in the study, all office workers in participating buildings were emailed a Movement at Work Survey (accessible at: www.activebuildings.co.uk) to complete anonymously. At the end of the survey, participants were given the option to provide their contact details to take part in an objective measurement arm of the study described below. Participants were eligible for inclusion if they were aged ≥18 years, had no condition that limited movement, and did not work from home for ≥25% of their work time. Ethical approval was provided by UCL Ethics Committee (Reference 4400/001), and all participants provided informed and written consent. Data are from the Active Buildings study, whose authors may be contacted through http://www.activebuildings.co.uk/. Qualified researchers can request to access the data by emailing Dr Abi Fisher at abigail.fisher@ucl.ac.uk.

### 2.1. Primary Outcomes: Workplace Step Count and Sitting Time

Physical activity (workplace step count) and sedentary time (sitting) were measured objectively using the activPAL inclinometer. The activPALs were provided to participants in the workplace by trained researchers. They were fitted to the middle of the thigh of the dominant leg by waterproof adhesive dressing (allowing for wear during bathing and water-based activities) and worn all day and night for up to seven days. The activPAL has been successfully used in studies of office workers and has been validated for step count and time spent sitting [25]. Participants were considered to have provided valid data if the device had been worn for at least 3 consecutive workdays [26,27]. Participants were asked to complete a log book recording workplace arrival and departure times, bed and wake times and any periods that the device was removed. Daily work times were calculated from arrival times rounded up to the nearest hour (e.g., 09:30 to 10:00) and departure times rounded down (e.g., 17:30 to 17:00) to allow for settling into the building and reaching workstations and to ensure commuting time was not captured. Time-stamped data were summarised in 15 s intervals and analysed in hourly intervals (e.g., mean hourly averages). 

### 2.2. Primary Exposure: Spatial Variables

**Figure 1:**
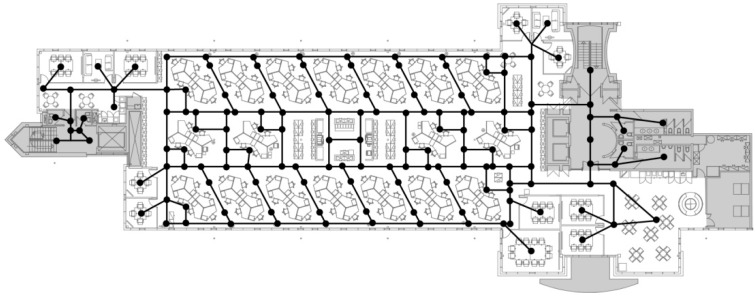
Example of a spatial graph.

A key deliverable of the Active Buildings study was to develop novel spatial variables to quantify spatial layout in office buildings objectively. Detailed description of the development of the spatial variables is provided as a technical appendix in Supplementary File 1. A tool for researchers to calculate these variables using office floor plans from any building has been developed and is freely available here: www.activebuildings.co.uk/spatialmetriccalculator. Spatial graphs were devised representing the possible circulation routes between individual workstations and key office destinations (i.e., key destinations: kitchens/coffee points, shared printers/copiers, meeting rooms, lifts, stairs, and WCs; Figure 1).

From the spatial graph, four metric categories were calculated. The first metric category was ‘distance from each workstation to key office destinations’, which was the shortest path from each participant’s workstation to each of the key office destinations. Two types of variables were generated, measuring length in metres, and in ‘edges’ (segments of the spatial graph). Participants reported perceived distance, as described in previous Active Buildings literature [23], which was significantly correlated with measured distance in metres (*r* = 0.271, *p* = 0.005). The second metric category was ‘distance from participant’s workstation to all other workstations on the floor’ (again in metres and edges). Third, ‘visibility of co-workers’ was assessed as how many co-workers were potentially visible within a 360-degree visual field from the participant’s workstation when the participant is either standing or sitting, after taking account of visual barriers such as solid walls and partitions. Visibility was computed from the spatial graph, to which visual barriers were added. Details of such barriers were derived from researchers’ observations during a site audit. Finally, ‘closeness’ was calculated to capture how ‘close’ a workstation is to all other points (i.e., nodes) on the spatial graph. Closeness was measured as the shortest distance in terms of number of turns or angular deviation and was devised to capture aspects of ‘connectivity’ and ‘integration’ in Duncan et al. [20] and to minimise changes in directions, as described in Sailer and McCulloch [28].

### 2.3. Covariates

Covariates were sociodemographics (age, sex, ethnicity, job role) from self-report in the Movement at Work Survey. Participant body weight was measured without shoes and in light clothing using Tanita electronic scales, and height was measured using a Stadiometer with the Frankfort plane in the horizontal position, by trained research assistants, from which body mass index (BMI) was calculated in kg/m^2^. Organisation was included as a covariate (descriptives of gross spatial metrics of organisations included in the Active Buildings study are shown in Table 1). In addition, in a previous Active Buildings analysis, perceived management discouragement of breaks was inversely strongly associated with workplace step count [23], so this was included.

### 2.4. Analyses

Some demographic data were missing at random and proportions of missing data were low (Table 2), so imputation was conducted using sample means (although results were the same with and without imputation). *T*-tests and chi-squares were used to assess demographic (age, sex, job role, and organisation) differences between those with complete activity data and without valid activPAL data; no differences were reported therefore there was not deemed to be any selection bias. Workplace step count and sitting time were not normally distributed. Data were log-transformed, but since results were the same whether using log-transformed or raw data, the raw data were presented as it was more meaningful to interpret. Linear regression models exploring associations between outcomes and exposures were calculated, then models were rerun after adjusting for covariates. Statistical significance was set at a *p*-value of <0.05. Analyses were performed using SPSS 22 (IMB Corp., Armonk, NY, USA).

## 3. Results

Of the 171 participants who provided activPAL data, 131 had ≥3 consecutive days of workday data. Participants with complete activPAL data were distributed between 10 organisations. These organisations differed in typology (e.g., cellular offices, open plan), geographical location (e.g., central London, suburban business park), and size (e.g., range in global metrics displayed in Table 1). There was a spread of organisational types: two were in the public sector, two in the private sector, and six in higher education. Seven organisations were contained within a single floor; three were spread across multiple floors (N.B. in these instances, key destinations were included on each of studied floors and spatial metrics were calculated for the floor on which the participant’s workstation was located).

Participant characteristics are provided in Table 2. Of the 131, 54% were female, 81% were white, and the majority reported that they had a managerial or professional role (71%). Participants had an average age of 39 years (SD 10.74) years and an average BMI of 25.64 (SD 4.49). The mean daily step count at work was 3412 steps (SD 1919) per workday and 444 steps (SD 210) per work hour. Participants spent an average of 5.46 h (SD 1.62) sitting per workday and 0.70 h (SD 0.15) sitting per work hour, equivalent to 42 min of every hour.

Associations between workplace stepping and spatial variables are presented in Table 3. There were significant negative associations between spatial variables and workplace steps per hour in simple and adjusted models. In adjusted models, as distance to all office destinations increased, steps per hour decreased (metres: B = −4.66, 95% CI: −8.12, −1.12, *p* < 0.01; turns: B = −9.08, 95% CI: −17.81, −0.35, *p* < 0.05). The same effect was revealed for distances to the nearest office destinations to the participant’s workstation (metres: B = −6.45, 95% CI: −11.88, −0.41, *p* < 0.05; turns: B = −17.32, 95% CI: −32.66, −1.97, *p* < 0.05). Visibility of other workstations when standing was associated with a decrease in step count; with every additional workstation, there was a decrease of two steps per hour (B = −2.35, 95% CI: −3.53, −1.18, *p* < 0.001). However, there was no effect for visibility of other workstations when standing (e.g., degree to which view was obstructed by mid-height partitions). Significant effects were very small for steps accrued per hour.

Table 4 displays associations between spatial variables and workplace sitting time per hour. No significant associations were reported in simple or adjusted models.

## 4. Discussion

This is the first study to explore associations between office layout and occupational physical activity using objective measures of both. Contrary to our original hypothesis, we found negative associations between distances to destinations and workplace step count. However, significant effects were small in magnitude; for example, for every metre increase in the mean distance to the nearest key destinations in the office, participants performed five fewer steps per hour. There were no significant associations between office layout and proportion of the work hour spent sitting. Small effect sizes and non-significant results could be viewed as consistent with results from a previous study from Active Buildings [23], which found no significant relationship between perceived distance and workplace step count, sitting, standing or sit-to-stand transitions. In contrast, perceptions of workplace culture (in particular, whether mangement were perceived to discourage breaks) were related to hourly workplace step count.

In the current study, objectively measured visibility of co-workers when standing was significantly associated with step count during the working day. However, the effect observed in our study was very small; for every additional workstation seen when standing at the workstation, two fewer steps per hour were accumulated. Using self-report to capture sitting time, Duncan et al. found that greater visibility of co-workers was associated with higher levels of sitting in a sample of 307 office-based workers in Australia [20]. Unlike those authors we did not find associations between distance-related spatial variables and sitting time. However, this may in part be explained by the different methodologies used: while Duncan et al. developed and used a self-report instrument and an established international questionnaire to capture environemntal variables and sitting, respectively, the current study included objective measures of both exposures and outcomes. Combined, the findings could indicate that visibility of co-workers (which could be a proxy for some other aspect of work) may have some impact on activity behaviour. However this hypothesis would need to be tested in larger samples with longitudinal or experimental study designs permitting some degree of causal inference. 

In addition, a negative association between distance to office destinations and step counts could indicate that participants in offices where destinations were more sparsely distributed accrued fewer steps because they reduced the number of trips taken from their workstation or because the distribution of destinations acted as a proxy for other elements of the interior environment or working culture. As noted, the magnitude of significant effects was small: although further research to unpick observed associations may elucidate underlying mechanisms, the value of focusing on small effect sizes must be weighed against research into other potential targets for environmental intervention in the workplace. Nonetheless, further research should assess whether the associations reported here are consistent in size and significance across different populations and/or building typologies.

Should the results reported here be replicated across different populations, building typologies, and organisations, there could be implications for policy and practice. For example, workplaces might endeavor to consider the visibility of co-workers and spatial layout of key destinations when designing new workplaces or when making modifications to existing office-space. However, it is not possible to confidently produce such recommendations without evidence of replicable effects and causal associations, which is not available from a single observational study.

To our knowledge, this is the first study to investigate the association between objectively measured spatial aspects of office buildings and objectively measured sitting and step count. The objective measurement of spatial variables, sitting time, and step counts are clear strengths of this study. Indeed, the development and definition of spatial variables that operationalise measurement of the office layout are beneficial in advancing this field. Operationalised spatial variables derived using the freely-available spatial metrics calculator developed in this study could help identify typologies of office buildings for future observational or quasi-experimental studies that further explore physical environmental influences on occupational physical activity and sitting time. Aside from these methodological strengths, the study does have limitations. The sample consisted predominantly of white office workers from London and the South East UK; it is not known whether these findings are representative of other groups. Given the intrusive nature of the study (which also included infrared tracking of individuals for analyses reported elsewhere [29]) it was relatively difficult to recruit companies and participants, therefore the organisation types recruited may not be fully representative. Furthermore, the spatial metrics focused on ‘horizontal’ movement within each floor and thus the impact of layout on ‘vertical movement’ via stairs was not explicitly considered. We focused on objective measures of the physical layout and only had minimal understanding of workplace activity policy through self-reported perceptions. Since this appeared to have a significant impact on workplace step count in a previous Active Buildings analysis [23], this is certainly an area for further exploration. For example, to the best of our knowledge, the organisations we studied did not have a specific policy to encourage movement and/or discourage prolonged sitting, and therefore we could not consider the combined effect of spatial layout and organisational policies around occupational activity through effect modification (e.g., moderation or mediation). Finally, the relatively small sample of organisations precluded examination of variation at an organisational, rather than individual, level, using techniques such as multilevel modelling. This would be an interesting focus for future research.

## 5. Conclusions

Data from this exploratory study suggest that increasing the relative distance between workstations and other office destinations within an office floor may not be the most fruitful target for interventions to increase physical activity and reduce sitting time for office workers. Further research is required to identify correlates that can be targeted to increase office workers’ physical activity and decrease sitting during the working day. Overall, the Active Buildings study has identified other potential targets for intervention including a focus on the journey to work and lunchtime movement [11] and considering workplace policy (e.g., perceived management allowance of breaks [23]), including its potential interaction with office layout.

## Figures and Tables

**Table 1:** Spatial characteristics of organisations in the Active Buildings study.

Metric	Mean	Range
Gross Internal Area (GIA; m^2^)	1418.06	535.99, 2978.12
Net Internal Area (NIA; m^2^)	1132.177	370.55, 2472.22
Net Useable Area (NUA; m^2^)	838.43	293.98, 2014.82
Number of workstations	123.48	52.00, 254.00
Gross Internal Density (m^2^/workstation)	12.15	7.68, 25.67
Net Internal Density (m^2^/workstation)	9.31	5.45, 18.24
Net Useable Density (m^2^/workstation)	6.80	4.56, 13.12

GIA: floor area measured from internal edge of perimeter walls of the building on each floor level. NIA: floor area measured from internal edge of perimeter walls of the building on each floor level, excluding area for structural walls or columns and vertical circulation, e.g., lifts, ducts for services. NUA: NIA excluding areas of circulation, escape routes and essential corridors. Density: floor area per person, where persons per floor is calculate as maximum number of people for whom the space has been furnished; can be expressed in relation to GIA, NIA or NUA.

**Table 2:** Participant characteristics.

Participant Characteristics (*n* = 131)	Mean (SD)	%
Age (years)	39.38 (10.74)	
Sex		
Male		42
Female		54
Missing		4
Ethnicity		
White		81
Non-white		15
Missing		5
Job role		
Professional-managerial		71
Telephone-administrative		22
Missing		7
Income (£)	31,244 (42,263)	
BMI	25.64 (4.49)	
Workplace physical activity and sitting time		
Step count/hour (steps)	444 (210)	
Step count/workday (steps)	3412 (1919)	
Sitting time/workday (hours)	5.46 (1.62)	
Sitting time/hour (hours)	0.70 (0.15)	
Spatial variables		
Distance office destinations (metres)	69.38 (15.09)	
Distance office destinations (edges)	14.04 (5.88)	
Distance nearest office destinations (metres)	39.38 (15.10)	
Distance nearest office destinations (edges)	14.04 (5.89)	
Distance workstations on floor (metres)	32.48 (15.06)	
Distance workstations on floor (edges)	12.50 (5.92)	
Visibility standing	45.96 (5.92)	
Visibility sitting	6.34 (5.00)	
Closeness (turns)	2.50 (0.72)	
Closeness (angular deviation)	233.76 (65.00)	

**Table 3:** Associations between spatial variables and workplace step count per hour.

	Simple Models			Adjusted Models		
	B	95% CI	*p*	B	95% CI	*p*
Mean distance to office destinations (excl. workstations)						
All destinations on floor (metres)	−4.36	−6.80, −1.92	**0.001**	−4.66	−8.12, −1.12	**0.009**
All destinations on floor (edges)	−9.77	−16.11, −3.43	**0.003**	−9.08	−17.81, −0.35	**0.042**
Nearest destinations (metres)	−7.80	−12.67, −2.93	**0.002**	−6.45	−11.88, −0.41	**0.036**
Nearest destinations (edges)	−21.20	−34.05, −8.35	**0.001**	−17.32	−32.66, −1.97	**0.027**
Mean distance to workstations on floor						
All workstations on floor (metres)	−3.29	−5.74, −0.822	**0.009**	−3.02	−6.53, 0.501	0.091
All workstations on floor (edges)	−7.42	−13.71, −1.13	**0.021**	−5.28	−13.94, 3.38	0.229
Visibility of other workstations						
Visibility standing	−2.26	−3.33, −1.16	**0.001**	−2.35	−3.53, −1.18	**<0.000**
Visibility sitting	−3.47	−11.07, 4.13	0.368	−4.48	−13.25, 4.24	0.314
Workstation ‘closeness centrality’						
Closeness (turns)	−62.40	−113.39, −11.39	**0.017**	−47.31	−102.65, 8.03	0.093
Closeness (angular deviation)	−0.44	−1.02, 0.14	0.132	−0.31	−0.94, 0.32	0.331

Simple models: univariate associations between IV (spatial metric) and DV (workplace step count per hour). Adjusted models: associations between IV, DV adjusted for organisation, participant age, sex, job role, BMI, ethnicity, perceived management discouragement of breaks. Bold typeface indicates significance at *p* < 0.05.

**Table 4:** Associations between spatial variables and minutes of workplace sitting time per hour.

	Simple Models			Adjusted Models		
	B	95% CI	*p*	B	95% CI	*p*
Mean distance to office destinations (excl. workstations)						
All destinations on floor (metres)	0.00	−0.001, 0.002	0.639	0.000	0.000, 0.001	0.092
All destinations on floor (edges)	0.002	−0.003, 0.006	0.534	0.001	−0.002, 0.003	0.568
Nearest destinations (metres)	0.000	−0.003, 0.004	0.855	0.000	−0.004, 0.005	0.866
Nearest destinations (edges)	0.004	−0.006, 0.014	0.446	0.004	−0.008, 0.016	0.512
Mean distances to all workstations on floor						
All workstations on floor (metres)	0.001	−0.001, 0.002	0.497	0.001	−0.001, 0.003	0.693
All workstations on floor (edges)	0.001	−0.003, 0.006	0.594	−0.004	−0.007, 0.006	0.989
Visibility of other workstations						
Visibility standing	0.001	0.000, 0.001	0.209	0.001	0.000, 0.001	0.129
Visibility sitting	0.004	−0.002, 0.009	0.202	0.003	−0.004, 0.009	0.448
Workstation ‘closeness centrality’						
Closeness (turns)	0.03	−0.008, 0.068	0.126	0.034	−0.007, 0.076	0.102
Closeness (angular deviation)	0.000	0.000, 0.001	0.158	0.002	−0.005, 0.008	0.641

Simple models: univariate associations between IV (spatial metric) and DV (workplace sitting time per hour). Adjusted models: associations between IV, DV adjusted for organisation, participant age, sex, job role, BMI, ethnicity, perceived management discouragement of breaks.

## Supplementary Materials

The following are available online at http://www.mdpi.com/1660-4601/15/6/1135/s1.
